# COVID-19 pandemic: the implications of the natural history, challenges of diagnosis and management for care in sub-Saharan Africa

**DOI:** 10.1186/s43088-021-00106-x

**Published:** 2021-03-17

**Authors:** Lawrence Omo-Aghoja, Emuesiri Goodies Moke, Kenneth Kelechi Anachuna, Adrian Itivere Omogbiya, Emuesiri Kohworho Umukoro, Pere-Ebi Yabrade Toloyai, Tarela Melish Elias Daubry, Anthony Taghogho Eduviere

**Affiliations:** DELSU Biomedical Research Alliance Working Group, College of Health Sciences, Delta State University, Abraka, Nigeria

**Keywords:** COVID-19, Natural history, Respiratory infection, Management, Nigeria

## Abstract

**Background:**

Coronavirus disease (COVID-19) is a severe acute respiratory infection which has afflicted virtually almost all nations of the earth. It is highly transmissible and represents one of the most serious pandemics in recent times, with the capacity to overwhelm any healthcare system and cause morbidity and fatality.

**Main content:**

The diagnosis of this disease is daunting and challenging as it is dependent on emerging clinical symptomatology that continues to increase and change very rapidly. The definitive test is the very expensive and scarce polymerase chain reaction (PCR) viral identification technique. The management has remained largely supportive and empirical, as there are no officially approved therapeutic agents, vaccines or antiviral medications for the management of the disease. Severe cases often require intensive care facilities and personnel. Yet there is paucity of facilities including the personnel required for diagnosis and treatment of COVID-19 in sub-Saharan Africa (SSA). It is against this backdrop that a review of key published reports on the pandemic in SSA and globally is made, as understanding the natural history of a disease and the documented responses to diagnosis and management is usually a key public health strategy for designing and improving as appropriate, relevant interventions. Lead findings were that responses by most nations of SSA were adhoc, paucity of public health awareness strategies and absence of legislations that would help enforce preventive measures, as well as limited facilities (including personal protective equipment) and institutional capacities to deliver needed interventions.

**Conclusion:**

COVID-19 is real and has overwhelmed global health care system especially low-income countries of the sub-Sahara such as Nigeria. Suggestions for improvement of healthcare policies and programs to contain the current pandemic and to respond more optimally in case of future pandemics are made herein.

## Background

### General information on COVID-19 and evolution of the disease

In 2007, Cheng et al. foresaw the likelihood of re-emergence of severe acute respiratory syndrome (SARS) infection which may be caused by SARS-coronavirus (CoV) or its congener, other novel viruses from animals or laboratories as a time bomb, and recommended the need for global preparedness [1]. This observation stemmed from the fact that there is the existence of a large reservoir of SARS-CoV-like viruses in horseshoe bats, coupled with the consumption of bizarre mammals, which is a cultural practice in specific provinces in China [1]. Little was it known that after the pneumonia epidemic caused by SARS-CoV in 2003 (which claimed over 800 lives [2]) and the Middle-East respiratory syndrome coronavirus (MERS-CoV) of 2013 (with a fatality rate of 50% [3, 4]), a novel coronavirus (SARS-CoV2) would hit the whole world in 2019/2020. The recently identified novel SARS-CoV2 is a member of the *Coronaviridae* family belonging to the subgenus, *Sarbecovirus* [5, 6]. The SARS-CoV2 ribonucleic acid (RNA) genome has been determined to be about 82% identical to SARS-CoV [7], but distinct from SARS-CoV and MERS-CoV [8, 9]. The SARS-CoV2 disease also christened coronavirus disease 2019 (COVID-19) is typically a severe acute respiratory infection caused by this novel virus [10]. This previously unfamiliar betacoronavirus was detected in the bronchoalveolar lavage specimens obtained from patients that presented with pneumonia of unknown cause [10, 11]. This virus was thus, acknowledged as the cause of pneumonia outbreak in Wuhan Metropolis, Hubei Province, China, in December 2019 [11].

The clinical presentation is that of a respiratory infection with signs and symptoms ranging from a slight common cold-like illness, to a severe viral pneumonia resulting in acute respiratory illness that is potentially deadly. The Chinese authority informed the World Health Organization (WHO) of this viral pneumonia on 31 December 2019. However, the majority of the index cases of COVID-19 were reportedly linked to the Huanan South China Seafood Market as the source of its origin [12]. The WHO announced that a novel coronavirus had been detected in samples taken from these patients and that laboratory tests ruled out SARS-CoV, MERS-CoV, influenza, avian influenza and other common respiratory pathogens as the causative agent [12, 13]. Since then, the outbreak has spread rapidly, with WHO first declaring COVID-19 a ‘public health emergency of international concern’ on 30 January 2020 and then formally declaring it a pandemic on 11 March 2020 [13]. The observed spread of this disease began in Wuhan, China, in December 2019 and is currently spreading to almost all parts of the world. The virus is transmitted from one individual to another or more persons via aerosol droplets which are expelled during coughing, talking or sneezing. Coronavirus infection may also be acquired by contact with aerosol droplets on infected surfaces and touching the nose, mouth or eyes. This is possible because the virus has the potential to remain viable on hard surfaces for a few days [14, 15]. Available evidence suggests that overcrowding that is common in low resource settings can serve as a catalyst for the transmission of the virus [16]. However, emerging reports from the USA tend to suggest that the spread of COVID-19 is limited by heat (high temperature), humidity and ultraviolet (sun) light [17].

### Symptomatology and clinical presentation

Basically, the virus perturbs the respiratory tract and within a space of 2–14 days, the individual affected manifests symptoms ranging from sore throat to dry non-productive cough and fever. Also, the illness caused by the virus is capable of causing confusion, malaise, headaches, dizziness and insensitivity to taste and smell. Based on these signs and symptoms, it is possible that the virus possesses the ability to upset the central nervous system (CNS), thus causing other CNS-related distress [18, 19]. This symptomatology is daily being updated by the WHO and relevant Public Health Agencies around the world. For instance, there is evidence that children hitherto thought to be spared from serious COVID-19 complications can be affected by a rare and potentially fatal inflammatory disease linked to the virus [20–22]. Also reported from some parts of USA and Europe are cases of what has been named paediatric multi-system inflammatory syndrome (PMIS), with some children experiencing organ failure and with a few deaths documented in the New York region [21]. It is believed that the virus triggers excessive immune reaction which results in widespread inflammation in the body of such children [21]. The patients may present with high-grade fever lasting 4 days or more, a rash, very red eyes, abdominal pain and skin peeling on hands or feet. The ensuing clinical condition is akin to Kawasaki disease, a rare childhood illness with similar signs and symptoms and may result in the enlargement of blood vessels that in severe forms may cause heart damage [20, 21]. Emerging evidence suggests that it has a significant impact on the cardiovascular (CV) system by direct myocardial damage, severe systemic inflammatory response, hypoxia, right heart strain secondary to ARDS and lung injury and plaque rupture secondary to inflammation [23]. Reported primary cardiac manifestations include acute myocarditis, myocardial infarction, arrhythmia and abnormal clotting [23].

### Detection techniques, treatment versus situation in SSA

Understanding COVID-19 case definition is very key and central to the diagnosis and treatment of this disease entity, which becomes very compelling in SSA with limited resources in terms of facilities, human resources and available funds to confront the disease. This will help to streamline the scope for the patients being assessed, and to chart a definite pathway for evaluation and treatment. In particular, the determination of a systematic algorithm will assist in sieving out patients that will require further detailed testing of the very expensive and scarce polymerase chain reaction (PCR) viral identification technique, from those that need to be self-isolated. To date, the case definition is based on the WHO report [24] from the global surveillance of human infections caused by COVID-19. Those tested are then classified into various categories—*suspected*, *probable*, *confirmed* and *asymptomatic cases*. In each of these four categories, the individual concerned should have a positive history of travel to or resident in a place with community transmission of COVID-19 within a 14-day period preceding the onset of symptom [14, 24]. The treatment is also quite challenging as there are no certain and approved therapeutics. A couple of agents and supportive treatment including intensive care facilities especially in severe cases have been deployed. But, findings from different ongoing studies have consistently called to question some of the earlier considered important intervention and treatment modalities. For instance, the real core value of routine use of ventilators and hydroxychloroquine in the management of COVID-19 has been questioned in several related studies [25, 26].

Studies are currently ongoing to learn more about COVID-19, as its specific source or origin is yet unknown. Essentially, the disease may probably be a re-emerging communicable respiratory infection similar to the Spanish flu of 1918, but it differs in the sense that it is caused by a new strain of coronavirus which produces respiratory distress in humans [14, 15]. Currently, the confirmed cases of the disease are slightly above 6.93 million, with about 6% case fatality rate (as of 8 June 2020; Table 1) [27]. All indices clearly demonstrate that countries across the globe are losing lots of funds in an attempt to contain the escalating spread of COVID-19 [30]. Whilst the incidence of the disease is still rising and yet to peak in SSA, the effects are already taking an excruciating toll on the health care system, and, individual and national economies of SSA nations. The sustainability of current interventions in these countries is debatable and threatening to collapse even in leading nations like Nigeria, Ghana and South Africa. Therefore, a review of documented reports globally and within SSA countries describing experiences of the patterns of the disease, its diagnosis and treatment become imperative. Understanding the natural history of a disease and the documented responses to diagnosis and treatment is usually a key public health strategy for designing and improving as appropriate, relevant interventions. The analysis in this paper examines the natural history of COVID-19, the global impact of COVID-19 particularly in developing countries of SSA, the emergence of covid-19 in SSA and the response so far, the challenges of diagnosis and treatment of COVID-19.

**Table 1:** COVID-19 situation updates worldwide, as of 14 August 2020 [27–29]

Location	Total confirmed cases	Total fatality (% of total)
African Region	1,085,589	24,680 (2.27%)
Region of Americas	11,291,921	411,129 (3.64%)
Asia	5,323,235	114,220 (2.14%)
Oceania	24,606	394 (1.60%)
European Region	3,174,730	208,928 (6.58%)
Other	696^a^	7 (1.01%)
Globally	20,900,777	759,358 (3.63%)

^a^Cases have been reported from an international conveyance in Japan

## Methodology

**Fig. 1:**
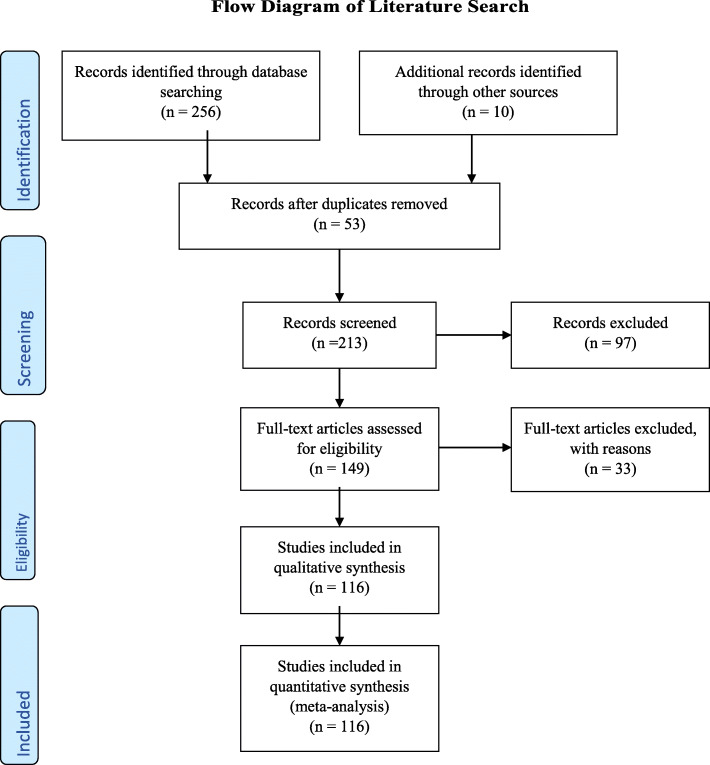
Flow diagram of literature search

The articles used for this review covered the period of 2005 to 2020, and in all 266 articles were retrieved following extensive literature search and of these, 116 were adapted for this article. Others were excluded either because the full texts of the articles were not retrievable or unavailable, or the scope and focus of the article were at variance with that of this review work. The papers adapted for this review article were either randomized controlled studies, cohort studies, case-control studies, clinical studies, case studies, case reports and cross-sectional studies. The extensive literature search undertaken was done using the following search engines or databases: MEDLINE, Elsevier, Medscape, eMedicine, Google and PubMed. The keywords that guided the literature search were COVID-19 pandemic, Natural History, challenges of diagnosis and treatment and SSA**.** Literature on the subject was also researched using manual library search from relevant cited textbooks and articles in journals, personal communication from respected frontline healthcare workers, and reports from several countries Centre for Disease Control (CDC) and the various task forces charged with the responsibility of coordinating the response to COVID-19 (Fig. 1).

## Main text

### Global impact of COVID-19 and implications for SSA

The COVID-19 pandemic all over the world has been associated with substantial morbidity and mortality (see Table 1), and as of 18 April 2020, there were total 2,121,675 confirmed cases globally, and an overall mortality rate of 6.7% with case fatality worse in the USA, Spain and Italy [31]. Additionally, it has severely tasked and overwhelmed the health care system even in developed nations of the West including America, UK and Europe which are bastion of healthcare delivery in the world [32]. The highly contagious nature of the virus and the attendant rapid spread of COVID-19 led to lockdown of countries as global public health strategy aimed at curtailing the virus, this was quickly adopted by nations of SSA including Nigeria, Ghana and South Africa amongst others [32]. The attendant consequences of the lockdown were closure of schools, religious gatherings, sporting activities and a host of other events, as social distancing and minimal contacts between individuals have been recommended by WHO as a veritable guideline to minimize the spread. The unforeseen partial or total lockdown took a heavy toll on businesses and economic activities particularly in SSA nations where a large proportion of business and trading activities are for subsistence of individual and families with little or no social support system [32]. The world economies were not spared particularly in SSA with very weak systems, and even nations considered to have strong economic systems were also rapidly lapsing into recession [30–32].

It is instructive to note that available data indicates that a high proportion of the citizens of SSA live in absolute poverty, with income and food insecurity [33]. About 75% are inadequately housed living in overcrowded conditions and/or with unimproved water supplies and sanitation [33]. Thirty-two of 46 SSA countries are classified by the United Nations (UN) as ‘Least Developed Countries (LDC)’ [33]. SSA region has a large cluster of LDCs, and the limited resources available to these countries, as well as their low capacity and reach, affects their ability to respond efficiently and effectively to health emergencies. Of the 46 countries in SSA, 18 has been classified as fragile and/or conflict-affected states by WHO, and this constitutes half of all such classifications globally [33].

Forty-one of the 46 SSA countries are in the bottom 5th of countries worldwide ranked by healthcare access and quality. Governments of several of the countries in SSA have invested and continue to invest poorly in healthcare depending substantially on donor assistance, prepaid plan and out-of-pocket payments. Many governments in the region have underinvested in healthcare, relying heavily on pre-paid plans, out-of-pocket payments and donor assistance. The exception being South Africa that approaches the medical workforce threshold (4.45 doctors, nurses and midwives per 1000 population) that the WHO determines to be necessary for Universal Health Coverage; the median for the region is just 0.95 [33, 34].

Approximately 20% of confirmed cases of COVID-19 patients seen in SSA, are usually severe, requiring hospital admission, and 5% were ‘critical’ that needed intensive care. Available data indicates that there are huge differences in the available hospital bed spaces between countries in SSA and those in the European Economic Area. Though clear-cut information on intensive care facilities is not available, but they are certainly scarce. Low- and middle-income countries as in SSA have around 0.1–2.5 beds/ 100,000 population, compared with a range of 5 to 30/100,000 population in high-income countries [33]. Secondary and, particularly, tertiary care facilities are inequitably distributed, as well as being concentrated in major cities. Most of the population in SSA is largely rural and underserved as typically seen in as much as 27 of the countries in the region. This clearly shows that life-saving critical care resources will be quickly overwhelmed even by much smaller outbreaks than have been seen elsewhere [33].

Despite above mentioned disturbing indices to be especially concerned about the outbreaks developing across SSA, the world’s attention was directed largely and essentially on the efforts to control transmission in China and on the evolving emergencies in Europe and North America. Yet SSA with very limited capacity for effective clinical response barely merited concerns despite the perfect storm of COVID-19 transmission brewing in the region.

The pandemic has continued to stir up panic in the minds of most people in the world because of the inability to accurately define its risk factors, coupled with the ability of the virus to overcome the immunological system of humans especially in the elderly and individuals with co-morbidities [7, 14, 35]. This is worse in SSA with very elementary public health measures to reach out to those with the known risk factors, a large pool of undiagnosed individuals with the co-morbidities that helps propagate COVID-19 and the poor health-seeking behaviour of the populace of SSA countries. The burden of the disease is certainly much higher across sub-Saharan Africa than in other world regions, particularly amongst children and young adults. This especially so as the primary drivers (chronic infectious disease such as TB and HIV, and undernutrition) which weaken immunity and make people more vulnerable to COVID-19 infection and more likely to develop severe illness or die, are widespread in countries of this region [32, 36]. COVID-19 is also likely to wreak havoc upon the delivery of established, prioritized but fragile essential healthcare programmes [32].

Whilst the WHO attempts to set policy and has recommended strategies to control the virus, there is yet a lot of variability in the responses of individual countries to the pandemic. The responses have been driven by the state and availability of healthcare personnel and infrastructure in the countries, the ability to implement extensive testing and to implement public health strategies given their varied socio-economic status. This notwithstanding, the global response is founded upon a number of measures which include rapid case identification; isolation and treatment; and the use of primary prevention techniques. Primary prevention includes improved personal hygiene with frequent hand washing and the use of hand sanitisers, physical and social distancing and the use of protective face masks. However, the available data so far reveals that the effects of the virus in more advanced countries such as the USA and UK have been more devastating than in less developed countries [27, 28, 31]. The isolates found in Africa are different from what is observed in Europe and the United States and have reduced virulence of SARS-CoV-2. So, speculation could be that the lower mortality rate from COVID-19 in Africa is due to genetic factors [37, 38]. Although the number of COVID-19 cases and fatalities might still appear comparatively low in SSA than in other world regions, the looming health shock of COVID-19 could have disastrous impacts on the continent’s already strained health systems, and could quickly turn into a social and economic emergency [32]. Beyond health risks, the COVID-19 shock to African economies is coming in three waves: firstly, lower trade and investment from China in the immediate term; secondly, a demand slump associated with the lockdowns in the European Union and Organisation for Economic Co-operation and Development (OECD) countries; and thirdly a continental supply shock affecting domestic and intra-African trade [39].

As shown in Table 1, the confirmed cases and the death toll so far recorded in region of the Americas, Europe and Asia is presently far higher than the available records in the African continent. Some reports have suggested that the higher numbers seen in more advanced countries are due to the higher testing rates in those countries [14, 22]. It is however worth noting that most of these countries reached the peak of their infections before countries in Africa, the reason for the delayed spread and peak in Africa is still the subject of much speculation. Whatever the case, the potential devastation COVID-19 brings demands that all countries provide adequate infrastructure, equipment and personnel to fight this disease. The worldwide scramble for medical equipment such as ventilators, personal protective equipment (PPE) and testing kits amongst others implies that demand will skyrocket and far out-weigh supply. Obviously, countries with low purchasing power, especially those in SSA and South America will be in serious distress [40–43].

Besides, it is high time low resource nations including SSA begin to look inwards and figure out how they can use their available financial and human resources to produce these essential items needed to combat the disease. In this moment of unimagined crisis, healthcare workers and the equipment they need to do their work have become highly paramount in coping with COVID-19 as countries struggle to ‘flatten the curve’ of the rate of its spread. It is becoming clearer that there is a need to actively support front line health-care team by re-strategizing work schedules so that the health of vulnerable older and health-compromised workers is not jeopardized at this time of acute health crisis [14].

### The emergence of COVID-19 in SSA and the response so far

The 46 countries of SSA region is home to well over one billion people (14% of the world’s population). The first confirmed case of COVID-19 infection in the region was reported, in Nigeria, on 28 February 2020, just 2 months after the first notification of a pneumonia of unknown cause in Wuhan, China [33]. The index case of COVID-19 in Nigeria was a 44-year Italian businessman who travelled to Lagos and Ogun states for a business trip in late February 2020 [43, 44]. The Director General of the World Health Organization, Dr Tedros, subsequently went on to classify the COVID-19 outbreak as a global pandemic on 11 March 2020. By 1 April 2020, 43/46 SSA countries had reported confirmed cases of COVID-19 (see Table 2). All of the 46 SSA countries have since reported cases. Table 3 shows the COVID-19 core indicators for SSA countries, by region (to August 8, 2020) [45]. The data on SSA regional incidence of COVID-19 has Southern Africa coming first with a wide margin to Eastern Africa which comes second, Western Africa occupies the third position and Central Africa with the least incidence [45]. The Southern African region has the highest case fatality rate of 1.8% which is similar to the overall case fatality rate for all regions put together, and the other three regions (West, East and Central) have same case fatality rates of 1.7%, respectively. However, the overall real extent of the pandemic remains conjectural, as cases are underreported and the means of data collection are subject to varying degrees of accuracies. Initially, the cases were confined to country capital cities, but cases are now reported are in multiple provinces with community spread actively ongoing [32, 39].

**Table 2:** Epidemiology of COVID-19 cases in Africa as of 18 April 2020 [32, 39]

Country	Confirmed cases	Deaths	Recoveries	First case/s
Angola	19	2	6	21 March 2020
Benin	35	1	18	16 March 2020
Botswana	15	1	0	30 March 2020
Burkina Faso	557	35	294	9 March 2020
Burundi	6	1	4	31 March 2020
Cameroon	1106	21	168	6 March 2020
Cape Verde	56	1	1	20 March 2020
Centr. Afr. Republic	12	0	5	14 March 2020
Chad	33	0	8	19 March 2020
Comoros	0	0	0	NA
Congo-Brazzaville	143	6	11	10 March 2020
DR Congo	307	25	26	10 March 2020
Equatorial Guinea	79	0	3	14 March 2020
Eritrea	35	0	0	20 March 2020
Eswatini	19	1	8	14 March 2020
Ethiopia	96	3	15	13 March 2020
Gabon	108	1	7	12 March 2020
Gambia	9	1	2	17 March 2020
Ghana	641	8	83	12 March 2020
Guinea	477	3	59	13 March 2020
Guinea-Bissau	50	0	0	25 March 2020
Ivory Coast	742	6	220	11 March 2020
Kenya	246	11	53	12 March 2020
Lesotho	0	0	0	NA
Liberia	76	7	7	16 March 2020
Madagascar	117	0	33	20 March 2020
Malawi	17	2	0	2 April 2020
Mali	190	13	34	25 March 2020
Mauritania	7	1	2	13 March 2020
Mauritius	324	9	108	19 March 2020
Mozambique	34	0	2	22 March 2020
Namibia	26	0	4	14 March 2020
Niger	627	18	110	19 March 2020
Nigeria	493	17	159	27 February 2020
Rwanda	143	0	65	14 March 2020
Sao Tome and Principe	4	0	0	6 Aprril 2020
Senegal	342	3	198	2 March 2020
Seychelles	11	0	5	14 March 2020
Sierra Leone	26	0	0	31 March 2020
South Africa	2783	50	903	5 March 2020
South Sudan	4	0	0	5 April 2020
Tanzania	147	5	11	16 March 2020
Togo	83	5	48	6 March 2020
Uganda	55	0	20	20 March 2020
Zambia	52	2	30	18 March 2020
Zimbabwe	24	3	2	15 March 2020

**Table 3:** COVID-19 core indicators for SSA countries, by region (to August 8, 2020) [45]

Indicator	All countries	Western	Central	Eastern	Southern
New cases	10,742	1069	291	2025	7566
Cumulative cases	843,322	164,405	31,851	89,633	557,433
Attack rate (per 100,000)	77.82	37.19	23.53	21.13	679.07
New deaths	365	16	5	32	312
Cumulative deaths	14,884	2717	538	1521	10,108
Case fatality rate	1.8%	1,7%	1.7%	1.7%	1.8%

In December 2019, the Africa CDC and Prevention in response to COVID-19 launched the Pathogen Genomics Intelligence Institute. The overall desired result of the institute is setting up a network of laboratories across the continent with capacity to genome sequence. The view then was that countries without the ability to sequence genomes can send their samples to one of the regional labs [46]. Equipment, software and reagents needed to sequence the virus was donated to the tune of $1.4 million by Illumina through the Africa CDC, to 10 countries on the continent at the end of April 2020, to equip them with the tools to perform next-generation sequencing. It committed to supporting these countries for a year [47]. It is instructive to note that laboratories in the Democratic Republic of the Congo, Gambia, Ghana, Nigeria, Senegal, South Africa and Uganda have been involved in sequencing of genomes of SARS-CoV-2.

The Africa CDC and Prevention in order to oversee Africa’s readiness for and to ensure adequate response to the global pandemic of the 2019 Novel disease, established the Africa Task Force for Novel Coronavirus (AFCOR) which comprised five working groups: (a) surveillance, including screening at points of entry; (b) infection prevention and control in healthcare facilities; (c) clinical management of persons with severe 2019-nCoV infection; (d) laboratory diagnosis and subtyping; and (e) risk communication and community engagement [48]. The European Centre for Disease Prevention and Control (ECDC) further partnered with Africa CDC in this regard, and this helped to reinforce the capacity of Africa CDC to respond to these public health threats across SSA and also expedited coordinated surveillance and disease intelligence, which tremendously supported the implementation of the public health workforce strategy of Africa CDC [49]. With this in place, the Africa CDC working in conjunction with national CDC in respective SSA countries, as well as their Ministries of Health (MOH) [50] went into action immediately intensifying, first, public awareness and stepping up possible prevention strategies in a bid to curtail its spread throughout the region and country. The vast majority of SSA countries put in place containment measures similar to those implemented by OECD countries, from closing their land borders to complete country lockdowns. All returning travellers from other countries as well as their primary contacts were informed to self-isolate for 14 days. The messaging on good personal hygiene such as frequent hand washing over running water for at least 20–30 s, the use of alcohol-based hand sanitizer, wearing of face mask and maintenance of physical distance of at least 2 m of person to person, was communicated to the public [28]. There was also the focus on interstate and intrastate border and travel restrictions within countries, school closures, bans on large gatherings, and case isolation. Existing testing facilities in various parts of the country were identified, upgraded as necessary and accredited as testing centres, whilst a few new centres were opened by some state’s government. Basically, Nigeria for instance has about 30 functional molecular testing laboratories for COVID-19, with at least more than one in each of the geopolitical zones in the country and efforts are underway to get more laboratories [28]. Also, contact tracing of confirmed cases was instituted and the primary contacts of the confirmed case(s) quarantined.

There were policy responses in selected economies and the responses are highlighted in Table 4 below [39].

**Table 4:** Fiscal measures implemented by selected African countries [39]

Country	Fiscal measures
Côte d’Ivoire	The government adopted an emergency response plan of CFAF96billion (178 million USD or 0.3% of GDP). The government announced a package of CFAF 820 billion (USD 1.43 billion or 2.3% GDP) of economic measures to prop the income of the most vulnerable segments of the population through agricultural input support and expanded cash transfers, provide relief to hard-hit sectors and firms, and support public entities in the transport and port sectors to ensure continuity in supply chains.
Ethiopia	Ethiopia initially announced a Br 300million (USD 8.76 million) package to bolster healthcare spending in early March. On 3 April, the Prime Minister’s office announced a COVID-19 Multi-Sectoral Preparedness and Response Plan, with prospective costing of interventions. The plan is to be implemented over the following months and will require USD 1.64 billion in funding (about 1.6% GDP).
Nigeria	A fiscal stimulus package in the form of a COVID-19 intervention fund of N500 billion (USD 1.4 billion), has been approved by the President to support healthcare facilities, provide relief for taxpayers, and incentivize employers to retain and recruit staff during the downturn. A presidential task force (PTF) on COVID-19 was constituted and saddled with the responsibility of meeting and briefing the nation on the day-to-day steps and efforts geared towards containing and curtailing the virus. PTF also make recommendations to the President on the modalities on how to further curtail the spread of the disease which is now in the community transmission phase.
Senegal	The government has set up an emergency fund of up to CFAF 1000billion (USD 1.74 billion, 7% of GDP), financed by a mix of donor contributions, voluntary donations from the private sector, and the budget. The Fund will be used to support vulnerable households and firms.
South Africa	The government is assisting companies and workers facing distress through the Unemployment Insurance Fund (UIF) and special programmes from the Industrial Development Corporation. Additional funds are being made available for the health response to COVID-19, workers with an income below a certain threshold will receive a small tax subsidy during the next four months, and the most vulnerable families will receive temporarily higher social grant amounts for the next six months. A new 6-month COVID-19 grant was also created to cover unemployed workers that do not receive grants or UIF benefits and the number of food parcels for distribution was increased. Funds are available to assist Small and medium-scale enterprises (SMEs) under stress
Uganda	The authorities have used part of their Contingency Fund in the FY2019/20 budget to finance approximately 1/5 of the Ministry of Health Preparedness and Response Plan from January to June 2020 (about USD 1.3million from a total of USD 7million). The government has passed a supplementary budget of about USD 80 million to support critical sectors such as health and security at the frontline of this pandemic. The government is working closely with the private sector and other stakeholders on measures to stimulate the economy following the COVID-19 pandemic.

### Challenges associated with sample collection and Diagnosis of COVID-19

The symptoms of COVID-19 - fever, cough, difficulty breathing and muscle pain - can resemble those of many other diseases, such as malaria and influenza which are widespread in SSA, making diagnostic tests therefore essential for identifying people who actually have COVID-19, as the symptoms cannot for obvious reason be relied upon [51]. Although fever and cough were the most common symptoms, extra-pulmonary manifestations especially digestive symptoms were the major complaint noted in a number of COVID-19 patients [51, 52]. In the study by Wang et al. 14 cases out of 138 (10.1%) hospitalized COVID-19 patients had initial digestive symptoms of diarrhoea and nausea, then fever and dyspnoea [53]. And, a set of 6 cases from a cohort of 204 patients with COVID-19 were found to have only digestive symptoms, without respiratory symptoms [54]. Asymptomatic patients and patients with other atypical symptoms such as loss of sense of smell or taste also have been reported [55, 56]. These findings have increased the uncertainty of the diagnostic work-up and raised concerns amongst clinicians. Moreover, given the lack of effective vaccines or treatments for COVID-19 until now, early identification of persons infected with SARS-CoV-2 virus followed by proper quarantine are the essential means to control the global pandemic.

Samples for testing are usually collected under appropriate infection prevention and control procedures guidelines which include proper handling of swab and use of PPEs [27, 57]. The utilization of the infection prevention and control procedures guidelines in SSA is usually poor. The probable, none utilization of PPE may be due to scarcity or increased general global demand, and the non-deployment of appropriate biosafety techniques in sample handling as a prevention measure is due to poor knowledge of biosafety practices amongst personnel who man healthcare facilities and laboratories in SSA [57]. Therefore, biosafety is a major concern in the developing world as the mishandling of biomaterial has the potential to evoke another episode of pandemic [58, 59]. The WHO recommends that all the requirements of Biosafety Level-2 (BSL-2) or their equivalent are met at facilities handling COVID-19-related specimens and performing non-propagative molecular testing [60, 61].

Essentially, a respiratory tract sample is obtained from patients with COVID-19 within 5 to 6 days of the manifestation of symptoms when they are believed to have a high load of the virus in their respiratory system [58, 62, 63]. The World Health Organization (WHO) has recommended nasopharyngeal swabs (NP), oropharyngeal swabs (OP), and nasopharyngeal or endotracheal washes as upper respiratory specimens in ambulatory patients [64, 65]. NP specimens are usually collected because this method of collection is relatively less invasive and it has been reported that the SARS-CoV-2 RNA is more detectable in NP as relative to OP [66]. Lower respiratory specimens include sputum, endotracheal aspirate, and bronchoalveolar lavage. Sputum production relies on productive coughs from patients. Asymptomatic or pre-symptomatic patients may be unable to produce sputum. Another challenge of collecting lower respiratory specimens is the risk of exposure to SARS-CoV-2 for personnel collecting specimens because of the requirement for close-contact with potential COVID-19 patients and irritation of respiratory airways during sampling. On the other hand, the OP swab sample collection process may stimulate a gag-like reflex, but person to person variability does exist in this response. As a result, these methods of sample collection may discourage patients from taking the test, hence other means of collecting upper respiratory tract specimens will be needed [57, 67]. One alternative option for obtaining an upper respiratory tract sample is a self-collected saliva sample [57, 68]. In the event, that the supply of swabs become scarce, self-collected saliva or nasal washes may be the only specimens available, and if not done properly, the sample might miss SARS-CoV-2 RNA when subjected to testing. Better still, SSA countries have to resort to local manufacturing of swabs, in order to achieve steady sample collection activity. It is worthy of note, that in some circumstances, both NP and OP swab may miss the detection of early infection, and repeated testing or obtaining lower respiratory tract samples might be necessary [67].

Quick detection and rapid diagnosis of COVID-19 are crucial in order to prevent its transmission and provide supportive care in a timely manner for patients. To enable accurate diagnosis, it should be noted that the clinical presentation of COVID-19 resembles viral pneumonia and that the severity of illness varies from mild to severe. However, it has been reported that roughly 80% of patients present with mild ailment, 14% present with severe sickness, and 5% present with severe ailment [14, 69]. Severe illness due to COVID-19 has been shown to be linked with older age and the existence of other underlying health conditions that may be life-threatening [14, 69]. Individuals with comorbidities as well as elderly persons could present with mild symptoms but are more at higher risk of deterioration [70]. It should be noted that the elderly or patients who are immunocompromised, particularly may present with atypical signs and symptoms of the COVID-19. Therefore, all patients should essentially be assessed in accordance to the pneumonia severity indices and sepsis guidelines, if sepsis is suspected and their samples collected (blood and sputum) to rule out other causes of lower respiratory tract infection and sepsis, especially patients with an atypical epidemiological history. These Samples have to be collected prior to the starting of empirical antimicrobials, as the case may be [14, 70].

Molecular testing, using real-time polymerase chain reaction (RT-PCR) is required to confirm the diagnosis of infection with COVID-19. The diagnostic tests are performed in accordance with the guidelines issued by local health authorities and adherence to appropriate biosafety practices. In a case where testing is not available in certain countries of SSA or regions, states or cities of specific countries, samples are shipped to the nearest accredited reference laboratory of neighbouring countries, region or states. Unfortunately, most of the countries still has few laboratories in the network of COVID-19 testing reference molecular laboratories accredited by the Africa CDC or respective country CDC. This may be responsible for the low number of total tests conducted so far as well as the reported cases in most of the SSA countries including Nigeria and Ghana as compared to some of the developed nations like the USA [27]. In SSA the Republic of South Africa takes the lead in testing so far for COVID-19 [26]. The performance of a nucleic acid amplification test, such as RT-PCR, for the detection of SARS-CoV-2 RNA is the approach that has been adopted by countries like Nigeria presently as the diagnostic tool for COVID-19. This technique is good but it can be time consuming (takes about 50 h) [71], thus resulting in the delay of test results as compared to the use of the Genexpert machine, which can produce test results much faster (takes less than 2 h) [72]. Also, the scarcity and increasing demand for test kits and other required reagents globally for effective molecular testing, is an obvious challenge stirring at the nations of SSA. Additionally, there is lack of trained manpower capable of performing the molecular biology experiments (e.g., viral RNA extraction and qPCR) required to test for SARS-CoV-2 and interpreting the results is another major limitation in the testing and confinement of COVID-19 in developing countries [61]. These are some of the major challenges, bedevilling the overall testing output of SSA, as the disease requires active testing and case search, more so when the disease infection mode has currently transformed from the realm of imported cases to community-based transmission.

There is emerging evidence [73–77] that lung ultrasound may be a useful aid in the diagnosis of COVID-19 as it has high sensitivity for detecting pleural thickening, subpleural consolidation, and ground-glass opacity. It has the advantages of portability, bedside evaluation, reduced healthcare worker exposure, and repeatability during follow-up. Characteristic ultrasound patterns have been reported in patients with COVID-19 and include B-lines, white lung, pleural line thickening, and consolidations with air bronchograms [73–77]. However, it also has limitations, in that it is unable to discern chronicity of a lesion and other imaging modalities may be required. Even at that, the gradual spike in the number of cases would limit the use of this diagnostic tool. There is also paucity of the facility and trained personnel with the technical knowhow to operate and perform specialized ultrasonography of this scope to contend with in SSA nations.

Researchers have also used computed tomography (CT) scanning to diagnose COVID-19, demonstrating good diagnostic sensitivity (97%) relative to RT-PCR [61, 78]. This veritable tool which comes handy in making prompt diagnosis, rapid isolation and management of patients, also has inherent problems that limit its applicability in SSA nations for the diagnosis of COVID-19. Firstly, there is paucity of capacity required to provide the level and scope of cleaning and hygiene needed in the CT-suites that will minimize contamination between examination. This is accentuated in face of the limited resources available in poor countries in SSA, and more importantly, there is escalation of the potential for cross-infection of COVID-19 negative patients during scanning from contaminated machines. Secondly, trained and skilled imaging Physicians who are needed to perform, examine and interpret these scans are in severely short supply in developing countries. Thirdly and probably most important, is the extreme paucity of CT scan machines for the reason of the high acquisition cost and the cost to undertake the investigations on patients.

There is an ongoing exploration of the use of biomarkers in the prognostication and follow-up of COVID-19 patients. Examples of such biomarkers include serum troponin, d-dimer and inflammatory markers such as C-reactive protein (CRP), white cell count (WCC), interleukin-6 (IL-6), lactate dehydrogenase (LDH), platelet count and renal markers (serum urea, serum creatinine) [53, 79–98]. Serum levels of these biomarkers are not used primarily for diagnostic purposes but rather for assessment of the disease severity and progression, as rising levels of these biomarkers are usually indicative of worsening severity of the disease condition.

In many ways, COVID-19 diagnosis underscores the main difference between ‘analytic’ and ‘clinical’ sensitivity, which is, the capability of an assay to detect pathogen that are present in any clinical sample as against the ability of the test to identify an individual’s general disease status. Factors such as sample-location and method of collection, together with the viral load as a function of anatomic location, disease severity, and time symptomatic as well as individual variability may also influence diagnosis.

### Challenges associated with treatment of COVID-19 patients

Despite the highly effective mode of transmission and comparatively low mortality rate of COVID-19 [19], the disease presently stands as the greatest global public health challenge in recent times with dire need of treatment. To date, no definite treatments are known to be effective for COVID-19 so far; for this reason, the mainstay of management is early detection, optimisation of supportive care to relieve symptoms, and the maintenance of organ function as much as possible in more severe cases of the disease. The effective management of COVID-19 is somewhat challenging as the use of supportive therapies cannot be over-emphasized, because definite cure still remains elusive. The use of fluids to conservatively manage patients with severe acute respiratory infection, with no evidence of shock, should be with care, as aggressive fluid resuscitation may worsen oxygenation [70]. Complications such as ARDS, sepsis, and septic shock should be managed according to usual protocols [14, 70]. Also, the use of empirical antimicrobials to cover other potential bacterial pathogens that may cause respiratory infection must be based on the clinical diagnosis [71]. Some patients with severe infection may require continued antimicrobial therapy once COVID-19 has been confirmed depending on the clinical circumstances. Besides, current evidence does not support routine antipyretic administration to treat fever in acute respiratory infections [89]. It has been suggested that non-steroidal anti-inflammatory drugs (NSAIDs) like ibuprofen might worsen COVID-19 or have an undesirable impact on disease outcome [90]. Otherwise, if necessary, NSAIDs could only be administered whilst symptoms are present, but caution is advised. Based on current information, the recommended medication of choice for the management of pyrexia in COVID-19 patients is paracetamol [90–94]. However, those that present with cough symptoms are advised to avoid lying on their back as this makes coughing ineffective. Also, the use of simple measures like administration of a teaspoon of honey may be considered first, before opting for the short-term use of opioid, if the cough is distressing to the patient [91].

In the case of managing breathlessness, the room must be kept cool, patients are to be relaxed and occasional changing of body positions should be encouraged to aid proper breathing. Also, the use of an opioid and benzodiazepine combination in patients with moderate to severe breathlessness or patients with breathing distress maybe appropriate, but if breathing distress is identified as reversible, the option of an oxygen therapy may be considered, if available [91]. Furthermore, patients should be monitored closely for signs of clinical deterioration, such as rapidly progressive respiratory failure and sepsis, so as to immediately start general supportive care interventions as appropriate [70]. Unfortunately, there are limited numbers of ventilators, which is necessary to prevent respiratory failure in most low resource countries in SSA that can cater for the available increasing number of patients that require it, and this may result to inevitable fatality of patients with severe case of the COVID-19 [22, 70]. So, there is the need to look inwards and encourage the manufacturing of locally made ventilators which will be far cheaper and efficient, as paucity of this equipment continues to subsist globally. It is disturbing to note that some citizens across the country still conceive the notion that COVID-19 is a disease of the elite and wealthy, due to the fact that the earlier index cases in the country were imported cases, whilst some believed it simply does not exist or that they are immune to it. This mind set may cause some persons not to comply with treatment regimen and threaten caregivers to release them from isolation, whilst still being positive with COVID-19. Also, religious and cultural believes coupled with self-medication are identified factors that prevent some individuals with suspected case of COVID-19 to present themselves for testing. This may be responsible for a number of fatalities which defiled optimisation of supportive care, as at the time of presentation of the cases in question, to the health facilities.

Nevertheless, the continued change in treatment modality, e.g. the ventilators that were once thought to be lifesaving are indeed now thought not to be the most appropriate modality for handling the respiratory distress in severe COVID-19 case, especially in some patients with underlying comorbidities. As recent observations suggest that acute respiratory distress syndrome may not be the only contributory factor for the development of respiratory failure seen in severe COVID-19 cases [92], but that pulmonary infarction, like disseminated intravascular coagulation, venous thromboembolism and pulmonary embolism, may also play a role. This may have important implications for the diagnostic and therapeutic management of COVID-19 disease [92]. Thus, the proposition that anticoagulants might be useful in the management of COVID-19. However, some drugs have been identified and listed for solidarity trials. As of but 23 May 2020, one of the arms of the trial with hydroxychloroquine was temporarily halted based on concerns raised about the safety profile of the drug, but on 3 June 2020, the decision was reversed by the WHO’s Director-General and recommended the continuation of all arms of the solidarity trial, including hydroxychloroquine [93]. Other drugs that have been used in different centres across SSA and beyond are remdesivir and dexamethasone [94]. The place of remsdivir is however uncertain as the WHO has issued a conditional recommendation against the routine use of the drug in hospitalized patients, regardless of disease severity, as there is currently no evidence that remdesivir improves survival and other outcomes in these patients. However, suppressing the hyperinflammation-associated COVID-19 disease with corticosteroids was shown to be efficacious at reducing mortality in the RECOVERY trial, with the greatest benefit amongst those requiring mechanical ventilation (mortality 29.3% in the dexamethasone group *vs* 41.4% in the usual care group; RR 0.64, 95% CI 0.51–0.81) [94]. The pressure that COVID-19 puts on the health systems has led to the consideration and need to speed up the trials. In normal palace, randomized clinical trials take years to design and conduct, but the solidarity trial will reduce the time taken by approximately 80% and may help facilitate the rapid worldwide comparison of unproven treatments [95]. It is worthy of note that getting and enrolling volunteers in multistage randomized trials can be very challenging due to lack of willingness on the part of some patients as well as the bureaucracies and compromise within the health care systems in low resource settings [95, 96]. Also, the technical knowhow for the production of vaccines is currently lacking in Africa, coupled with scepticism and reservation people in low resource nations have for vaccines use [97]. Excitedly, vaccines have now been produced, approved and released for public use in the USA and the UK. Currently, two vaccines are authorized and recommended to prevent COVID-19: Pfizer-BioNTech COVID-19 vaccine and Moderna's COVID-19 vaccine. It is recommended that only one type of COVID-19 vaccine is received. For the two approved vaccines, two doses are required and both doses must be of the same brand of vaccine. Report shows that utilization and uptake of these vaccines in SSA would be largely determined by the perceived efficacy and safety from utilization in the developed nations of the West. A survey conducted by the Africa CDC in partnership with the London School of Hygiene and Tropical Medicine across 15 African countries to investigate public knowledge and perceptions about the COVID-19 pandemic and COVID-19 vaccine revealed that a predominant majority (79%) of respondents in Africa would vaccinate against COVID-19 if it was deemed safe and effective [98].

Another area of major challenge in the management of COVID-19 is the use of unproven traditional herbal medicine often with uncertain mechanism of action in the treatment of patients with COVID-19 disease. A large proportion of the global population make use of traditional herbal medicine [99, 100]. It has been reported that over 80% of the people in the developing world such as in SSA patronize the use of medicinal plants in the treatment of ailments [101]. The World Health Organization (WHO) welcomes inventions around the world including repurposing drugs, traditional medicines and developing new therapies in the quest for potential treatments for the novel COVID-19 infection [102]. Presently, there are claims of the effectiveness of traditional herbal medicine in the therapy of COVID-19. These herbal medicines range from traditional Chinese Medicine to African Herbal remedies. Ang et al. wrote a review on the use of herbal medicines and pattern identifications for the treatment of patients with various stages of COVID-19 infection (Chinese and Korean guidelines) [103]. Likewise, the antiviral potential of some Chinese herbal remedies has also been reported [104, 105].

With African governments struggling to contain the COVID-19 pandemic, very small health budgets, inadequate health infrastructures, and too few health personnel pose to be the major setbacks for the success of this struggle [61]. This has steered many African countries into sourcing for local herbal remedies for COVID-19 therapy. Amongst these African herbal remedies is the ‘COVID-Organics’, an herbal tonic produced in Madagascar, which contains *Artemisia annua* (Sweet wormwood) plant often used to treat malaria. The World Health Organization (WHO) has stated that it is critical to establish the efficacy and safety of medicine of natural products through rigorous clinical trials [100], and is, thus, yet to approve the use of *Artemisia annua*. The proof of efficacy and safety as prerequisite for approving and recommending any medication by WHO is underscored by the fact of the upsurge in the incidence of COVID-19 in Madagascar despite the consumption of COVID-Organics leading to a total lockdown. Another herbal remedy is the “cancer bush plant” (*Sutherlandia frutescens* L.), a South African herb traditionally used to treat a variety of health conditions including internal cancers, diabetes, and a variety of inflammatory conditions [106–109]. The deficit of the well-documented safety profile of herbal medicine and the lack of clinical trials on traditional herbal drugs is a major drawback for its use globally [101, 102]. Inspite of these known facts, their use as first-line medications by a significant proportion of the populace in SSA has continued to challenge the treatment of COVID-19 disease with attendant late presentation and consequent high probability of fatality of such cases.

Finally, a challenge with contact tracing and the confounding effects of certain intervening disease conditions that usually cause a delay in seeking care in SSA poses further challenge to the diagnosis and treatment of COVID-19. Contact tracing is central and key to the effective and efficient management of COVID-19, and the inability to effectively undertake this is a huge challenge to containing and curtailing the propagation of COVID-19. Effective contact tracing in the setting of most SSA nations is herculean and daunting as the persons exposed may not likely present themselves voluntarily for isolation and testing, and those positive cases may deliberately not reveal all persons they have been in contact with. The terrain and numbering of cities and communities also pose difficulty in tracing of contacts of index cases. Disease conditions that have potential confounding effects on the diagnosis of COVID-19 infection include malaria and other infections/infestations that are endemic in SSA with capacity to cause systemic perturbations of the human body particularly the respiratory system [110]. The COVID-19 illness is akin to the clinical picture of these patients, posing a diagnostic and treatment challenge [44, 50, 61].

## Conclusion

Based on the recommendations by the Africa CDC, National CDC and respective MOH of SSA countries, patients should be managed in an accredited hospital setting (isolation centres) [28]. From available indices, there will be a likely challenge to this endorsement of isolating patients as available centres are rapidly being overwhelmed, when the pandemic still persists and peak still distant away. As the available healthcare infrastructures are becoming overwhelmed, many ethical questions arise as to how best to triage patients with a view to saving the most lives in the accredited health facilities across the countries of SSA. Recommendations have been suggested, but there are no clear international guidelines on this issue presently [111–115], let alone in low resource settings like SSA nations. It has also been observed that some of the frontline staffs are being infected with COVID-19, which may make other potential frontline staffs to drawback, except for those that are selfless and would not decline the Hippocratic Oath. As a result of these challenges, patients may progressively resort to alternative care homes, which may further put more people at risk of contracting the disease. This may continue, until a vaccine or approved drug for COVID-19 is produced because there is no definite effort by the government to sponsor drug discovery at the moment. Most low resource countries, especially in SSA is waiting for the developed world for permanent solution to this pandemic.

A comprehensive national and SSA regional response action plan and implementation for the fight against COVID-19 should be developed by the Ministries of Health that will be focused on the improved health of the entire citizenry. There should be more concerted efforts to increase public health awareness and educate the rural communities about the severity of COVID-19 through the National Orientation Agencies. Also, policy makers should develop appropriate policies and legislation that could protect the right of patients and survivors of COVID-19 from stigmatization. The government should encourage and regulate the practice of traditional healers and establish a working relationship between them and orthodox medical counterparts. Furthermore, the government should encourage and fund indigenous manufacturing of test swabs, kits, PPEs, ventilators and other accessories which will be far cheaper and efficient, as paucity of this equipment and materials continues to subsist globally. Also, the respective governments of SSA nations should fund and support researches that will lead to the development of local remedies, which will be certified by their National Agencies for Food Drug Administration and Control (NAFDAC) that could be of potential benefits for the treatment of COVID-19 amongst other infections.

## Data Availability

Not applicable.

## Abbreviations

ARDS: Acute respiratory distress syndrome
CDC: Centre for Disease Control
COVID-19: Coronavirus disease
CT: Computerized tomography
GDP: Gross Domestic Product
LDC: Least Developed Countries
MERS-CoV: Middle-East respiratory syndrome coronavirus
MOH: Ministry of Health
NAFDAC: National Agency for Food Drug Administration and Control
NSAIDs: Non-steroidal anti-inflammatory drugs
OECD: Organisation for Economic Co-operation and Development
PCR: Polymerase chain reaction
PMIS: Paediatric multi-system inflammatory syndrome
PPE: Personal protective equipment
RNA: Ribonucleic acid
RT-PCR: Real-time polymerase chain reaction
SARS: Severe acute respiratory syndrome
SARS-CoV: Severe acute respiratory syndrome coronavirus
SSA: Sub-Saharan Africa
UIF: Unemployment Insurance Fund
WHO: World Health Organization
